# The extreme insular adaptation of *Garganornis ballmanni* Meijer, 2014: a giant Anseriformes of the Neogene of the Mediterranean Basin

**DOI:** 10.1098/rsos.160722

**Published:** 2017-01-11

**Authors:** Marco Pavia, Hanneke J. M. Meijer, Maria Adelaide Rossi, Ursula B. Göhlich

**Affiliations:** 1 Dipartimento di Scienze della Terra, Museo di Geologia e Paleontologia, Università degli Studi di Torino, Via Valperga Caluso 35, 10125 Torino, Italy; 2 Department of Natural History, University Museum, University of Bergen, Postboks 7800, 5007 Bergen, Norway; 3 Soprintendenza archeologia, belle arti e paesaggio dell'Abruzzo, Via degli Agostiniani 14, 66100 Chieti, Italy; 4 Department of Geology and Paleontology, Natural History Museum Vienna, Burgring 7, 1010 Vienna, Austria

**Keywords:** fossil bird, Anseriformes, flightlessness, insular gigantism, Miocene, Italy

## Abstract

New skeletal elements of the recently described endemic giant anseriform *Garganornis ballmanni* Meijer, 2014 are presented, coming from the type-area of the Gargano and from Scontrone, southern and central Italy, respectively. The new remains represent the first bird remains found at Scontrone so far, and another shared element between these two localities, both part of the Apulia-Abruzzi Palaeobioprovince. The presence of a very reduced carpometacarpus confirms its flightlessness, only previously supposed on the basis of the very large size, while the morphologies of tarsometatarsus and posterior phalanges clearly indicate the adaptation of *G. ballmanni* to a terrestrial, non-aquatic, lifestyle. Its very large body size is similar to that observed in different, heavily modified, insular waterfowl and has been normally interpreted as the response to the absence of terrestrial predators and a protection from the aerial ones. The presence of a carpal knob in the proximal carpometacarpus also indicates a fighting behaviour for this large terrestrial bird species.

## 1. Introduction

Since Darwin's and Wallace's works, the study of islands has been a powerful tool in many evolutionary studies, and birds are a key group in understanding the biological changes in island environments. Birds on islands often display dramatic size increases, allometric variations and, in some cases, flightlessness. The latter is known in different bird group like ibises, cormorants, rails, pigeons, parrots, passerines and waterfowl [1–4].

The waterfowl, crown-group Anseriformes, includes Anhimidae (South American screamers), Anseranatinae (Australasian magpie-geese) and Anatidae with a worldwide distribution [5]. The fossil record of Anatidae is very rich and comprises several endemic species, which evolved flightlessness in insular conditions, both on remote oceanic islands [4,6–8] as well as in intermediate-type islands, such as those of the Mediterranean Sea [9–11]. Most of the insular anatids overall show slight reduction of the wings towards flightlessness [7,12–14], but in some cases, they were extremely adapted to a terrestrial lifestyle with a great increase in body size, such as the Hawaiian moa-nalos *Chelychelynechen* and *Thambetochen* [4] and New Zealand's *Cnemiornis* [15]. Among the giant insular species of Anseriformes was *Garganornis ballmanni*, a species recently described from the Neogene fissure-filling deposits of the Gargano (southern Italy) on the basis of a single distal tibiotarsus [16].

**Figure 1.**
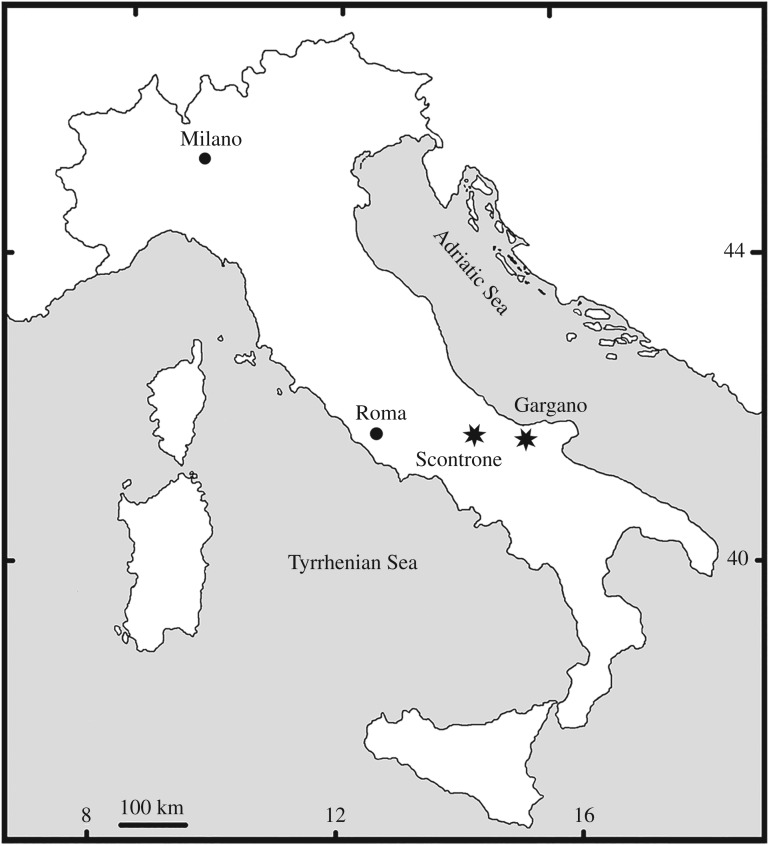
Map of Italy with the location of Gargano and Scontrone.

Here we describe new skeletal elements of *G. ballmanni* from Gargano and Scontrone, southern and central Italy, respectively (figure 1), the main localities of the so-called Apulia-Abruzzi Palaeobioprovince [17], which during the Miocene was characterized by insular conditions and a highly endemic fauna. These new remains allow us to clarify the peculiar adaptations of *Garganornis* to a terrestrial lifestyle and confirm its flightlessness, which was only previously suggested [16]. In addition, *G. ballmanni* represents the first fossil bird found at Scontrone, and a new terrestrial taxon shared between Scontrone and Gargano, showing that it was widespread in the Apulia-Abruzzi Palaeobioprovince.

## 2. Material and methods

### 2.1. Study areas

The Gargano area (Foggia, southern Italy) during the Late Miocene was an insular domain that was part of the Apulia-Abruzzi Palaeobioprovince [17]. In the sediment filling the karst network, exposed in the quarries exploiting the Mesozoic Calcare di Bari near the town of Apricena, a large amount of vertebrate remains has been found, demonstrating the high diversity of the fauna inhabiting this insular domain [18]. The Scontrone fossil locality is situated on Monte Civita, close to the village of Scontrone (L'Aquila, central Italy). The fossil remains have been found in the lower part of the Scontrone Calcarenite, marginal marine carbonates representing the early stage of the Tortonian transgression, in an interval represented by coastal sandbars [19]. Gargano and Scontrone represent the most important fossil localities of this palaeobioprovince [17], which was characterized by very peculiar vertebrate associations with highly endemic macro- and micromammals, reptiles and birds [16–18,20–25], all of them grouped in the so-called *Mikrotia* fauna [18,26]. It is worth mentioning that the fossil vertebrate association of this palaeobioprovince is also characterized by the absence of terrestrial predators, with the exception of the very peculiar hedgehog *Deinogalerix* [25], the otter *Paralutra* [27] and a crocodile [28], the latter two known from very few remains.

Despite the various common taxa of mammals and reptiles shared between Gargano and Scontrone, not even one taxon of the rich bird assemblage of Gargano has been found at Scontrone or in other localities of the palaeobioprovince until now [17]. This is most probably due to the taphonomic characteristics of the Scontrone deposits [29] and to the very hard sediments embedding the bones, which make it hard to find and collect the smaller and more fragile bones during excavations.

### 2.2. Methods

The fossil material is stored in the Dipartimento di Scienze della Terra of the Firenze University, Firenze, Italy (DSTF), the Museo di Geologia e Paleontologia of the Torino University (MGPT-PU), the Naturmuseum of Augsburg, Augsburg, Germany (NMA), the Naturalis Biodiversity Center, Leiden, the Netherlands (RGM) and the Centro di Documentazione Paleontologico *Hoplitomeryx*, Scontrone, Italy (SCT). The fossil specimens have been compared with the recent bird skeletons stored in the Laboratoire de Géologie de Lyon, Université Claude-Bernard Lyon 1, Lyon, France (FSL), the Dipartimento di Scienze della Terra of the Torino University, Torino, Italy (MPOC), the Natural History Museum, London and Tring, UK (NHMUK) and in the Ditsong National Museum of Natural History, Pretoria, South Africa (TM). The osteological terminology follows Baumel & Witmer [30]. The circumference of tibiotarsi of selected fossil Anseriformes at the National Museum of Natural History in Washington D.C., USA were measured by wrapping a thin strap of paper around the thinnest portion of the tibiotarsus and measuring the minimum circumference with sliding calipers calibrated to the nearest 0.05 mm. Body mass was then estimated using the least-squares regression estimates of Iwaniuk *et al*. [31].

## 3. Systematic palaeontology

Anseriformes Wagler, 1831

Anatidae Vigors, 1825

*Garganornis ballmanni* Meijer, 2014

(figures 2–4)

**Figure 2.**
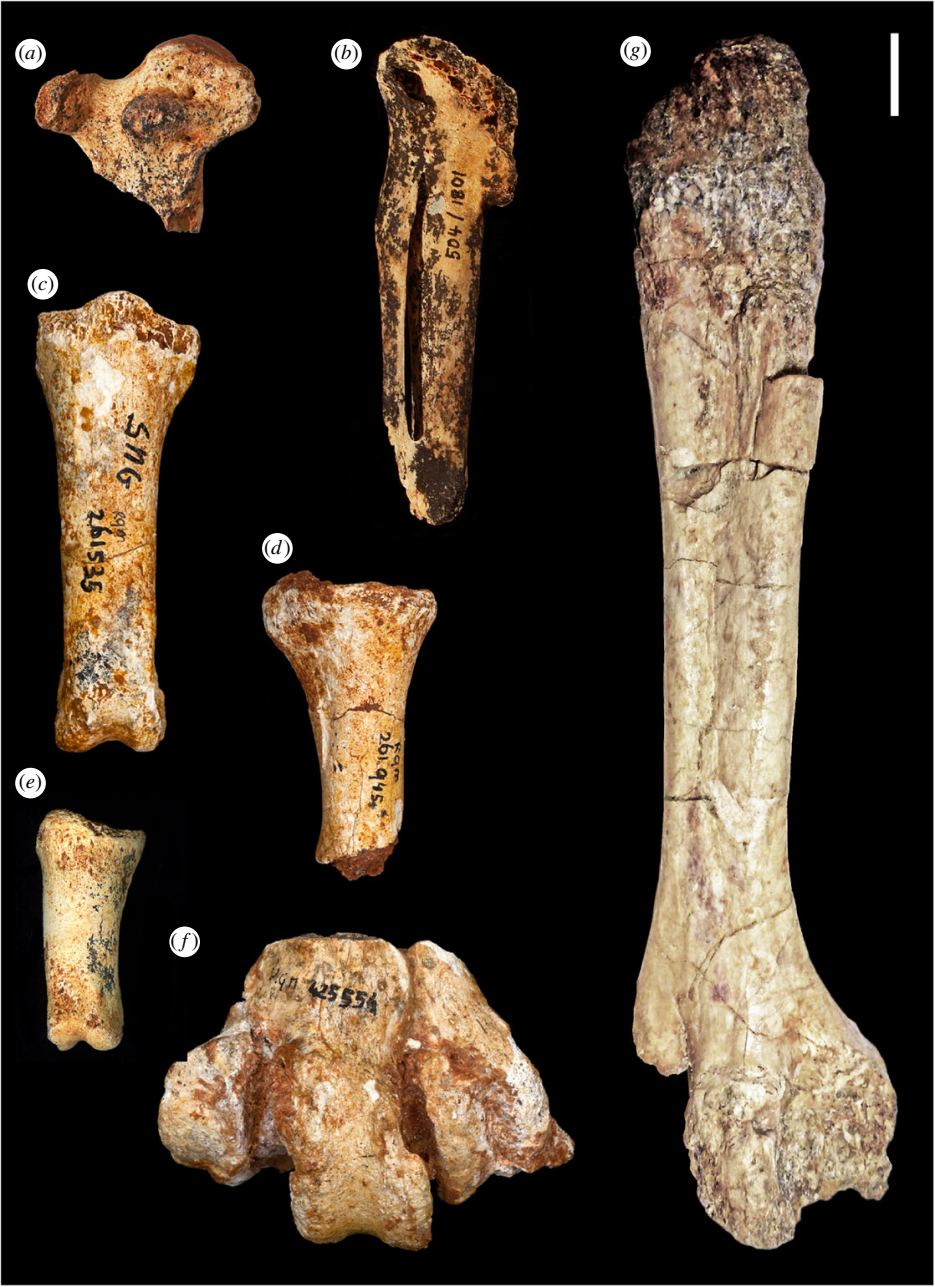
Bones of *Garganornis ballmanni* Meijer, 2014 from the Late Miocene of Gargano (*a–f*) and Scontrone (*g*), Italy. (*a*) Right carpometacarpus DSTF-GA 49, ventral view; (*b*) left carpometacarpus NMA 504/1801, ventral view; (*c*) proximal pedal phalanx RGM 261535, dorsal view; (*d*) proximal pedal phalanx RGM 261945, dorsal view; (*e*) mesial pedal phalanx MGPT-PU 135536, dorsal view; (*f*) left tarsometatarsus RGM 425554, dorsal view; (*g*) right tarsometatarsus SCT 23, dorsal view. Scale bar represents 1 cm.

**Figure 3.**
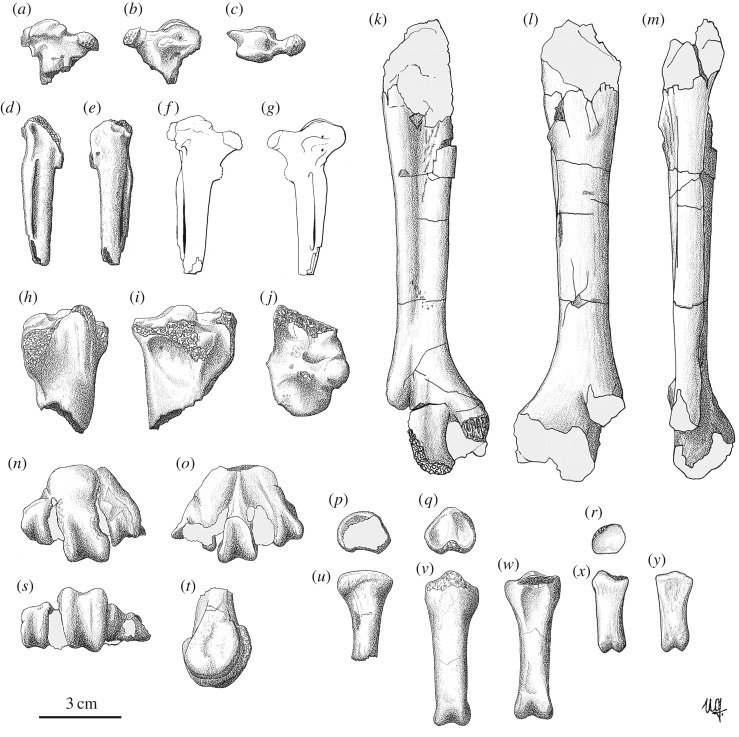
Drawings of the bones of *Garganornis ballmanni* Meijer, 2014; all the bones are from the Late Miocene of Gargano except one (*k*–*m*) which is from the Late Miocene of Scontrone, Italy. (*a–c*) Right carpometacarpus DSTF-GA 49 in ventral (*a*), dorsal (*b*) and proximal (*c*) views; (*d*,*e*) left carpometacarpus NMA 504/1801 in ventral (*d*) and dorsal (*e*) views; (*f*,*g*) graphical reconstruction of the carpometacarpus of *Garganornis ballmanni* based on the specimens DSTF-GA 49 and NMA 504/1801 in ventral (*f*) and dorsal (*g*) views; (*h–j*) right tibiotarsus DSTF-GA 77 in cranial (*h*), caudal (*i*) and proximal (*j*) views; (*k–m*) right tarsometatarsus SCT 23 in dorsal (*k*), plantar (*l*) and lateral (*m*) views; (*n,o*,*s,t*) left tarsometatarsus RGM 425554 in dorsal (*n*), plantar (*o*), distal (*s*) and medial (*t*) views; (*p*,*u*) proximal pedal phalanx RGM 261945 in proximal (*p*) and dorsal (*u*) views; (*q*,*v*,*w*) proximal pedal phalanx RGM 261535 in proximal (*q*), dorsal (*v*) and plantar (*w*) views; (*r*,*x*,*y*) mesial pedal phalanx MGPT-PU 135536 in proximal (*r*), dorsal (*x*) and plantar (*y*) views. Scale bar represents 3 cm. Drawings made by Ursula B. Göhlich.

**Figure 4.**
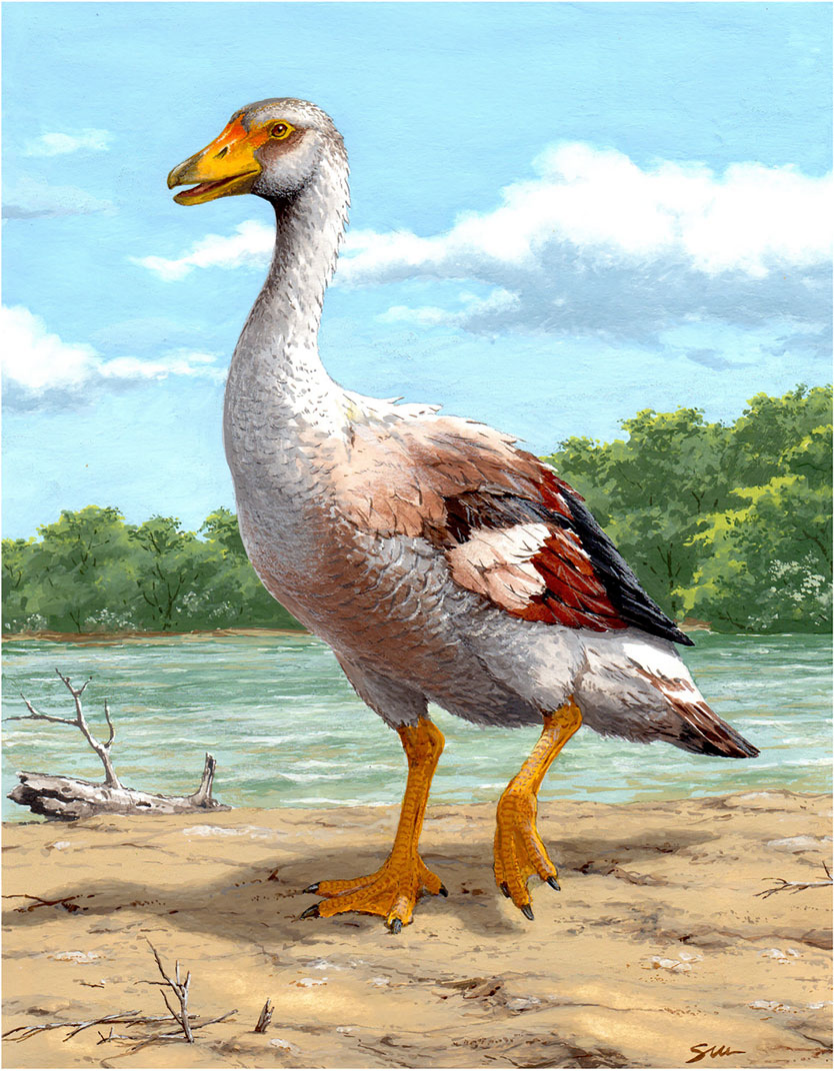
Reconstruction of *Garganornis ballmanni* Meijer, 2014 based on the newly described fossil remains. This reconstruction is based on a generic Western Palaearctic Goose with short and robust tarsometatarsus, short toes and very short wings according to the known elements of *Garganornis ballmanni*. Illustration made by Stefano Maugeri.

Holotype: distal left tibiotarsus RGM 443307 from the Late Miocene of Gargano (Posticchia 5), southern Italy.

New material from Gargano: right carpometacarpus DSTF-GA 49 (F15), proximal end; left carpometacarpus NMA 504/1801 (Fina 2), lacking proximal end; right tibiotarsus DSTF-GA 77 (F15), damaged proximal end; left tarsometatarsus RGM 425554 (San Giovannino), distal end; trochlea metatarsi III of a right tarsometatarsus RGM 425943 (Chirò 14A); indeterminate pedal phalanges MGPT-PU 135356 (unnamed fissure), RGM 261535 (Pizzicoli 12), RGM 261945 (unnamed fissure).

New material from Scontrone: right tarsometatarsus, SCT 23, almost complete with proximal and distal ends damaged and missing trochlea metatarsi IV.

Measurements: DSTF-GA 49: proximal width, 27.7 mm; proximal depth, 10.5 mm. NMA 504/1801: total length as preserved, 53.6 mm. DSTF-GA 77: proximal width, 43.2 mm; proximal depth, 31.0 mm (estimated). SCT 23: total length of preserved specimen, 168.2 mm; distal width, 22.5 mm (estimated); width of shaft at mid length, 18.3 mm. RGM 425554: distal width, 47.5 mm; distal depth, 24.8 mm (estimated). MGPT-PU 135356: total length, 29.0 mm; proximal width, 13.4 mm; proximal depth, 11.6 mm; distal width, 9.1 mm; distal depth, 7.7 mm; width of shaft at mid length, 9.9 mm. RGM 261535: total length, 55.1 mm; proximal width, 19.6 mm; proximal depth, 17.7 mm; distal width, 13.3 mm; distal depth, 11.9 mm; width of shaft at mid length, 11.3 mm. RGM 261945: proximal width, 20.9 mm; proximal depth, 16.2 mm; width of shaft at mid length, 9.7 mm.

Chronological distribution: *G. ballmanni* is now reported from the Tortonian locality of Scontrone, at an age of about 9 Ma [19], to the Late Miocene fissure fillings of Gargano, for which an age between 6 and 5.5 Ma has been recently proposed [32]. Across the karst network system of the Gargano area, the remains of *G. ballmanni* have now been found in different fillings spread over the various chronological phases outlined by Masini *et al*. [18,23] from the Phase 1b to the Phase 3c. In particular, the chronological attribution to the Phase 2b of the recently collected pedal phalanx MGPT-PU 135356 has been made after study of an associated *Mikrotia* specimen, a rodent of key importance to assess the relative ages of the samples from the Gargano fissure-filling system [18].

Comparative osteology: The proximal carpometacarpus DSTF-GA 49 (figures 2*a* and 3*a–c*) is very peculiar, with the two sides of the trochlea carpalis greatly extended laterally, rendering the proximal part of the bone very flat in respect to other Anseriformes. The presence of a processus extensorius parallel to the proximal trochlea and not tilted ventrally, a relatively wide and not pointed processus pisiformis, and a small knob dorsally to the fovea carpalis caudalis allows its attribution to the Anatidae. On the top of the processus extensorius of the proximal carpometacarpus DSTF-GA 49, we can observe a small bony outgrowth, similar in shape and size to that observed in other Anseriformes, in female *Pezophaps solitaria*, and in *Goura cristata* (Columbidae), but less developed than those of the male of *P. solitaria*, and surely not a spur as in Anhimidae, *Plectropterus* (Anatidae) and *Vanellus* (Charadriidae) [33]. Specimen NMA 504/1081 (figures 2*b* and 3*d–e*) lacks the proximal end, except the distal parts of the trochlea carpalis and the squared processus alularis. The shaft is very reduced in length with the spatium intermetacarpale very short, as demonstrated by the partially preserved synostosis metacarpalis distalis. Despite the distal end of the bone being incompletely preserved, the synostosis metacarpalis distalis is well developed, which indicates that the total length of the bone was not much longer than in the preserved specimen. The presence of a peculiar fossa on the ventral side of the carpometacarpus lateral to the processus pisiformis, which is also observed in other Anatidae although less evident, and other morphological details make it possible to graphically reconstruct the complete bone (figure 3*f–g*) and to confirm the relative shortness of the carpometacarpus. This is the first wing bone known for *G. ballmanni*.

The proximal tibiotarsus DSTF-GA 77 (figure 3*h–j*) is highly damaged but shows a very distinct fossa retropatellaris and a deep fossa flexoria, which are compatible with the morphology of Anatidae. The holotype tibiotarsus RGM 443307, already described in detail by Meijer [16], clearly shows its affinities with the Anatidae, rather than with other Anseriformes.

The tarsometatarsus SCT 23 (figures 2*g* and 3*k–m*), although badly preserved, is morphologically similar to the biggest Anatidae, but ever bigger than the extant *Cygnus cygnus* and the fossil *Cy. falconeri*, the giant extinct taxon from Sicily and Malta [34]. In particular, the tarsometatarsus is short and robust with a central hypotarsus, deduced from the distal part of the hypotarsus that is preserved, the sulcus flexorius absent, a well-marked sulcus extensorius, and a wide distal end with the rounded trochlea metatarsi III. The proximal end including the hypotarsus is not preserved. However, as the tuberositas musculi tibialis cranialis is visible on the specimen, the greatest length of the complete bone can be expected to be not much greater than the total length of the specimen. The crista plantaris lateralis is well developed, more protruding plantarly than in *Cygnus* and *Anser*, but similar to *Cereopsis*; the sulcus extensorius is very deep, deeper than in *Cygnus* and in the other large Anseriformes, and extends distally to the mid-point of the bone. The distal part of the diaphysis of the two fossil specimens is flat just proximal to the trochleae, which is not common in the Anseriformes, but similar to that observed in *Cereopsis*. The three trochleae metatarsi, preserved in the RGM 425554 (figures 2*f* and 3*n–q*), are latero-medially aligned in distal view with the trochlea metatarsi II and not tilted medially as in most of the Anseriformes. In addition, the trochleae metatarsi II and IV are more equal in distal extent than in most of the Anseriformes, but similar to what is observed in the Anhimidae, in *Cereopsis*, and in some unpublished specimens of the extinct *Cy. falconeri* preserved in the NHMUK (M.P. 2014, personal observation). It is important to note here that the arrangement of the distal trochlea differs from that in other large-bodied taxa such as cranes (Gruidae) and storks (Ciconiidae). The general morphology of the distal end of SCT 23 and RGM 425554 are very well comparable one to each other in both the shape of the preserved trochleae and in the flat area proximally to the trochleae. The Gargano avifauna does not contain any other birds of this size, and we therefore think it likely that both specimens represent the same species.

The pedal phalanges (figures 2*c–e* and 3*r–y*) are similar to those of the biggest Anatidae [15,34], but stouter with no strong ligament impressions.

Remarks: The faunal composition of the Apulia-Abruzzi Palaeobioprovince has been extensively studied [16,18,20–22,24]. The largest avian taxa are the Accipitridae *Garganoaetus freudenthali* and the Tytonidae *Tyto gigantea* [20,21]. Despite their size, their bones are smaller than the bones here described. In addition, as part of a revision of the whole bird association coming from the Palaeobioprovince, ongoing by one of us (M.P.), all specimens have been checked and all of the Anatidae remains have been published [24]. None of them is comparable in size with *G. ballmanni*.

The morphology of the bones described here, although highly modified, fits best with that of the Anatidae and their size is well compatible with *G. ballmanni*. Despite them coming from different localities, we refer them to *G. ballmanni*.

The study of the newly discovered material allows us to clarify the overall morphology of *G. ballmanni* and its adaptations to a terrestrial lifestyle. The morphology of the tarsometatarsus is different from most of the extant and fossil Anseriformes concerning the proportionately deeper and distally more developed sulcus extensorius, the dorso-plantarly flattened distal part of the shaft, and the distally and latero-medially almost aligned trochlea metatarsi II and IV. These features recall the tarsometatarsus morphology of Anhimidae and *Cereopsis*, among the more terrestrially adapted taxa within the crown-group Anseriformes. In addition, the preserved pedal phalanges are stout and short in respect to those of the swimming geese and swans.

Thus, the morphological characteristics of *G. ballmanni* reveal its adaptation to a terrestrial, non-aquatic, lifestyle and suggest that it probably lived in open or arid environments, as regularly observed in other strongly modified island Anseriformes [4,15].

## 4. Discussion

### 4.1. Taxonomic affinities

The analysis of the holotype tibiotarsus of *G. ballmanni* revealed its very large size, significantly bigger than *Cy. olor*. Some characters of the tibiotarsus are also seen in extinct lineages of Palaeogene and Neogene birds, such as Dromornithidae, Gastornithidae and Presbyornithidae. In particular, *G. ballmanni* shares with Gastornithidae a tibiotarsus with the wide fossa intercondylaris and the circular opening of the distal side of the canalis extensorius, but differs in lacking the strong sulcus m. fibularis and in having the distal end of canalis extensorius not positioned more medially as in Gastornithidae. However, the presence of these old taxa in the Neogene of southern Italy is unlikely, as there is no evidence of survival of Palaeogene faunal elements in the Apulia-Abruzzi Palaeobioprovince. Alternatively, and more likely, the peculiar morphologies shown by the holotype of *G. ballmanni* are the result of a convergent evolution of this species within an insular environment, where mammalian carnivores were extremely rare if not absent [25,27]. This theory is also supported by the morphology of the newly described bones, which are much more similar to Anatidae than to Gastornithidae and other stem-groups of Anseriformes. It is also worth noting that the carpometacarpus of *G. ballmanni* shows some similarity with the one of *Gastornis* sp. from Louvois (France), while it is very different from the one from Cernay (France) [35]. As with the holotype tibiotarsus [16], this similarity does not reflect any phylogenetic relationships between *Garganornis* and *Gastornis*, but rather indicates convergent evolution. The morphology of the new fossil remains, although strongly modified and incompletely preserved, combined with that of the holotype of *G. ballmanni*, confirms its attribution to the family Anatidae, the only crown-group Anseriformes recorded in the European Neogene.

The strong morphological modifications of *G. ballmanni* and the small number and poor preservation of the fossil remains do not allow for a hypothesis regarding its nearest continental non-endemic relatives among the Anatidae and the phylogenetic affinities of this endemic taxon.

### 4.2. Flightlessness and body mass estimation

In the original description of *G. ballmanni*, Meijer [16, p. 22] hypothesized that it was flightless on the basis of its very large size, compared with flying Anatidae. The newly described material, in particular the modified carpometacarpus, confirms this hypothesis and permits us to reconstruct the life appearance of *G. ballmanni* (figure 4). In fact, the estimated length of the carpometacarpus of *G. ballmanni* is proportionately much shorter than that of the extant large-bodied flying Anseriformes, and too shortened to allow the species to fly (figure 3*f–g*). In addition, the flattening of the proximal end and the lateral projection of the trochlea carpalis are unique features of *G. ballmanni* possibly related to a modification of the carpometacarpus following the loss of its flying ability. In fact, the carpal trochleae articulate the carpometacarpus with the ulna and the carpal bones and drive the extension and flexion of the wings. Their weakness in shape would indicate less movement of the wrist joint, as observed in the carpometacarpus of other flightless species [15,31,36]. In most of the flightless Anseriformes, the major wing modifications occur in the mid-wing elements, radius and ulna, rather than in the carpometacarpus [11]. The great reduction of the carpometacarpus, which is common in flightless Rallidae and Columbidae [2,4,37], was only recorded in the highly modified *Chendytes* [12], *Cnemiornis* [15], *Thambetochen* [4] and in the recently described *Shiriyanetta hasegawai* [7].

Flightlessness is most frequently interpreted as an adaptive response to the absence or reduced diversity of terrestrial predators. However, in the case of *G. ballmanni*, this taxon was still exposed to aerial predators, which were very abundant in the area during the Late Miocene [20,21]. The presence of large-bodied aerial predators, such as the eagle *Garganoaetus freudenthali* and the barn owl *T. gigantea*, may be one of the main reasons behind the great increase of the body size of *G. ballmanni*, together with the general tendency of insular big-sized Anatidae to become terrestrial herbivores. This is in contrast with insular flightless small-sized Anatidae which remain aquatic and show a reduction in body size [6,12]. On the basis of the minimum circumference of the holotype tibiotarsus (60.2 mm), Meijer [16, p. 22] already estimated the body mass of *G. ballmanni* at 22.3 kg, larger than any living anseriform. It should be noted that the minimum circumference of the holotype is not the minimum circumference of the bone itself, as the minimum circumference is located more proximally on the shaft. Adjusting for this, Meijer [16] instead gave a range estimate of 15.3–22.3 kg, at which *G. ballmanni* is still heavier than extant anatids. In fact, all the measurements of the bones of *G. ballmanni* that we could compare with recent and fossil big-sized Anatidae revealed its very large size, indicating that the real body mass is probably much closer to the maximum suggested by Meijer [16, p. 22]. Unfortunately, the newly described elements do not allow a new estimation, as the tarsometatarsus is involved in different functions in addition to the locomotion, such as feeding and perching, and therefore not a good indicator of body mass [31], and the wing elements are never considered in such analysis, in particular, those of flightless birds.

The large size of *G. ballmanni* becomes especially apparent in comparison with other insular wildfowls, as it is estimated to be one of the biggest Anseriformes to have ever existed (table 1). In particular, within the Mediterranean region, the extinct Maltese Swan *Cy. falconeri* also attained large size, and its size is estimated from 15.8 to 16.38 kg (mean of 16.09 kg) by Northcote [34]. The Hawaiian Islands were home to the large-bodied and flightless moa-nalo, but their estimated body sizes are well below that of *G. ballmanni* (table 1). The estimates for the New Zealand Geese *Cnemiornis* range from 8.03 to 15.55 kg (mean of 12.18 kg) for *Cn. gracilis,* and from 14.5 to 20.36 kg (mean of 17.46 kg) for *Cn. calcitrans* [8].

**Table 1.** Body mass estimates of *Garganornis ballmanni* Meijer, 2014 in comparison with other extinct insular Anseriformes.

species	location	age	body mass (kg)	source
*Garganornis ballmanni*	Gargano, Italy	Miocene	22.3	[16]
*Cnemiornis calcitrans*	New Zealand	Holocene	17.46 (14.5–20.36)	[8]
*Cygnus falconeri*	Malta	Pleistocene	16.09	[32]
*Cnemiornis gracilis*	New Zealand	Holocene	12.18 (8.03–15.55)	[8]
*Thambetochen chauliodous*	Hawaiian Islands	Holocene	7.9	this study
*Thambetochen xanion*	Hawaiian Islands	Holocene	6.4	this study
*Ptaiochen pau*	Hawaiian Islands	Holocene	5.9	this study

### 4.3. Fighting adaptation

In addition to its reduced length, the proximal end of the carpometacarpus of *G. ballmanni* shows evident morphological modifications of the processus extensorius. On the external side of the processus extensorius of the proximal carpometacarpus DSTF-GA 49, a small carpal knob is present (figure 2*a*). It is of small size and there is no evidence that it could have become a spur, as in Anhimidae, but it is similar to those observed in *Cygnus*, *Anser* and other Anatidae [38], including *Cy. falconeri* (M.P. 2014, personal observation based on undescribed material at NHMUK). The carpal spurs or knobs, when present, are almost exclusively located on the processus extensorius and are always used as weapons [33]. Many Anseriformes display wing spurs or knobs in connection with fight behaviour, such as Anhimidae, *Tachyeres*, *Chloephaga*, *Alopochen*, *Cereopsis* and other Anatidae [38]. The presence of this knob in *G. ballmanni* should therefore be correlated with wing fight behaviour.

## 5. Conclusion

The data presented here confirm the taxonomic validity of *G. ballmanni* as a strongly modified species of crown-group Anatidae endemic to the Late Miocene Apulia-Abruzzi Palaeobioprovince of central-southern Italy, on the basis of fossil remains found in the Gargano area and at Scontrone.

The newly described material, although not perfectly preserved, gives new osteological details of the morphology of *G. ballmanni*, which confirms its flightlessness and its extreme adaptation to a terrestrial, non-aquatic, lifestyle. Furthermore, we observed evidence for wing fighting behaviour.

*Garganornis ballmanni* also demonstrates the strong endemic character of the Late Miocene Apulia-Abruzzi Palaeobioprovince, already suggested by the highly modified mammals and birds [17]. The Mediterranean Sea thus confirms its role as centre of speciation and its islands can also be confirmed to be an intermediate type of island, with faunal composition halfway between oceanic and continental ones, characterized by a very impoverished mammal fauna with high degree of endemism and no terrestrial carnivores, and by a diversified bird fauna with highly modified taxa [11].

## Data accessibility

Our paper has no associated data or other supplementary material, other than that presented in the manuscript.

## References

[1] Cheke A, Hume JP 2008 Lost land of the dodo. The Ecological History of Mauritius, Réunion, and Rodrigues. New Haven, CT: Yale University Press.

[2] Feduccia AJ 1996 The origin and evolution of birds. New Haven, CT: Yale University Press.

[3] Newton I 2003 The speciation and biogeography of birds. London, UK: Elsevier.

[4] Olson SL, James HF 1991 Description of thirty-two new species of birds from the Hawaiian Islands: part I. Non-passeriformes. AOU Ornithol. Monogr. 45, 1–88. (doi:10.2307/40166794)

[5] Dickinson EC, Remsen JVJ 2013 The Howard & Moore complete checklist of the birds of the world, 4th edn Eastbourne, UK: Aves Press.

[6] Livezey BC 1989 Phylogenetic relationships and incipient flightlessness of the extinct Auckland Island Merganser. Wilson Bull. 101, 410–435.

[7] Watanabe J, Matsuoka H 2015 Flightless diving duck (Aves, Anatidae) from the Pleistocene of Shiriya, northeast Japan. J. Vert. Paleontol. 35, e994745 (doi:10.1080/02724634.2014.994745)

[8] Worthy TH, Holdaway RN 2002 The lost world of the moa: prehistoric life of New Zealand. Christchurch, New Zealand: Canterbury University Press.

[9] Alcover JA, Florit F, Mourer-Chauviré C, Weesie PDM 1992 The avifaunas of the isolated Mediterranean island during the Middle and the Late Pleistocene. Sci. Ser. Nat. Hist. Mus. Los Angeles County 36, 273–283.

[10] Mayr G, Pavia M 2014 On the true affinities of *Chenornis graculoides* Portis, 1884, and *Anas lignitifila* Portis, 1884—an albatross and an unusual duck from the Miocene of Italy. J. Vert. Paleontol. 34, 914–923. (doi:10.1080/02724634.2013.821076)

[11] Pavia M 2008 The evolution dynamics of the Strigiformes in the Mediterranean islands with the description of *Aegolius martae* n. sp. (Aves, Strigidae). Quat. Int. 182, 80–89. (doi:10.1016/j.quaint.2007.05.018)

[12] Livezey BC 1990 Evolutionary morphology of flightlessness in the Auckland Islands teal. Condor 92, 639–673. (doi:10.2307/1368685)

[13] Livezey BC 1993 Morphology and flightlessness in *Chendytes*, fossil seaducks (Anatidae, Mergini) of coastal California. J. Vert. Paleontol. 13, 185–199. (doi:10.1080/02724634.1993.10011500)

[14] Livezey BC, Humphrey PS 1986 Flightlessness in steamer-ducks (Anatidaea: *Tachyeres*): its morphological bases and probable evolution. Evolution 40, 540–558. (doi:10.2307/2408576) 10.1111/j.1558-5646.1986.tb00506.x 28556327

[15] Worthy TH, Holdaway RN, Sorenson MD, Cooper AC 1997 Description of the first complete skeleton of the extinct New Zealand goose *Cnemiornis calcitrans* (Aves: Anatidae) and a reassessment of the relationships of *Cnemiornis*. J. Zool. 243, 695–723. (doi:10.1111/j.1469-7998.1997.tb01971.x)

[16] Meijer HJM 2014 A peculiar anseriform (Aves: Anseriformes) from the Miocene of Gargano (Italy). C. R. Palevol. 13, 19–26. (doi:10.1016/j.crpv.2013.08.001)

[17] Masini F, Petruso D, Bonfiglio L, Mangano G 2008 Origination and extinction patterns of mammals in three central Western Mediterranean islands from the Late Miocene to Quaternary. Quat. Int. 182, 63–79. (doi:10.1016/j.quaint.2007.09.020)

[18] Masini F, Rinaldi PM, Petruso D, Surdi G 2010 The Gargano Terre Rosse insular faunas: an overview. Riv. Ital. Paleontol. Stratigrafia 116, 421–435.

[19] Patacca E, Scandone P, Carnevale G 2013 The Miocene vertebrate-bearing deposits of Scontrone (Abruzzo, Central Italy): stratigraphic and paleoenvironmental analysis. Geobios 46, 5–23. (doi:10.1016/j.geobios.2012.11.001)

[20] Ballmann P 1973 Fossile Vögel aus dem Neogen der Halbinsel Gargano (Italien). Scr. Geol. 17, 1–57.

[21] Ballmann P 1976 Fossile Vögel aus dem Neogen der Halbinsel Gargano (Italien). Zweiter Teil. Scr. Geol. 38, 1–59.

[22] Göhlich UB, Pavia M 2008 A new species of *Palaeortyx* (Aves: Galliformes: Phasianidae) from the Neogene of Gargano, Italy. Oryctos 7, 95–108.

[23] Masini F, Rinaldi PM, Savorelli A, Pavia M 2013 A new small mammal assemblage from the M013 Terre Rosse fissure filling (Gargano, South-Eastern Italy). Geobios 46, 49–61. (doi:10.1016/j.geobios.2012.10.003)

[24] Pavia M 2013 The Anatidae and Scolopacidae (Aves: Anseriformes, Charadriiformes) from the Late Neogene of Gargano, Italy. Geobios 46, 43–48. (doi:10.1016/j.geobios.2012.10.013)

[25] Villier B, van den Hoek Ostende LW, de Vos J, Pavia M 2013 New discoveries on the giant hedgehog *Deinogalerix* from the Miocene of Gargano (Apulia, Italy). Geobios 46, 63–75. (doi:10.1016/j.geobios.2012.10.001)

[26] Freudenthal M, van den Hoek Ostende LW, Martín-Suárez E 2013 When and how did the *Mikrotia* fauna reach Gargano (Apulia, Italy)? Geobios 46, 105–109. (doi:10.1016/j.geobios.2012.10.004)

[27] Villier B, Pavia M, Rook L 2011 New remains of *Paralutra garganensis* Willemsen, 1983 (Mustelidae, Lutrinae) from the Late Miocene ‘Terre Rosse’ of Gargano (Apulia, Italy). Boll. Soc. Paleontol. Ital. 50, 135–143. (doi:10.4435/BSPI.2011.13)

[28] Delfino M, Rossi MA 2013 Fossil crocodylid remains from Scontrone (Tortonian, Southern Italy) and the Late Neogene Mediterranean biogeography of crocodilian. Geobios 46, 25–31. (doi:10.1016/j.geobios.2012.10.006)

[29] Mazza PAP 2015 Scontrone (central Italy), signs of a 9-million-year-old tragedy. Lethaia 48, 387–404. (doi:10.1111/let.12114)

[30] Baumel JJ, Witmer LM 1993 Osteologia. In Handbook of avian anatomy: Nomina Anatomica Avium (ed. Baumel JJ), pp. 45–132, 2nd edn. Cambridge, MA: Nuttall Ornithological Club.

[31] Iwaniuk AN, Nelson JE, James HF, Olson SL 2009 A comparative test of the correlated evolution of flightlessness and relative brain size in birds. J. Zool. 263, 317–327. (doi:10.1017/s0952836904005308)

[32] Savorelli A, Colombero S, Masini F 2016 *Apatodemus degiulii* n. gen et sp. (Rodentia, Muridae), a hitherto undescribed endemite from the Terre Rosse of Gargano (Late Miocene, Southeastern Italy). Palaeontographica Abt. A Paleozool. Stratigr. 306, 25–49.

[33] Hume JP, Steel L 2013 Fight club: a unique weapon in the wing of the solitaire, *Pezophaps solitaria* (Aves: Columbidae), an extinct flightless bird from Rodrigues, Mascarene Islands. Biol. J. Linn. Soc. 110, 32–44. (doi:10.1111/bij.12087)

[34] Northcote ME 1982 Size, form and habits of the extinct Maltese swan *Cygnus falconeri*. Ibis 124, 148–158. (doi:10.1111/j.1474-919x.1982.tb03753.x)

[35] Mourer-Chauviré C, Bourdon E 2016 The *Gastornis* (Aves, Gastornithidae) from the Late Paleocene of Louvois (Marne, France). Swiss J. Palaeontol. 135, 327–341. (doi:10.1007/s13358-015-0097-7)

[36] Worthy TH, Mitri M, Handley WD, Lee MSY, Anderson A 2015 Osteology supports a stem-Galliform affinity for the giant extinct flightless birds *Sylviornis neocaledoniae* (Sylviornithidae, Galloanseres). PLoS ONE 11, e0150871 (doi:10.1371/journal.pone.0150871) 10.1371/journal.pone.0150871 PMC4814122 27027304

[37] Olson SL 1973 Evolution of the rails of the South Atlantic islands (Aves: Rallidae). Smithson. Contrib. Zool. 152, 1–53. (doi:10.5479/si.00810282.152)

[38] Woolfenden GE 1961 Postcranial osteology of the waterfowl. Bull. Fla State Mus. 6, 1–129. (doi:10.5962/bhl.title.104997)

